# Comparison of prognostic scores for alcoholic hepatitis: a retrospective study

**DOI:** 10.3325/cmj.2021.62.17

**Published:** 2021-02

**Authors:** Tonći Božin, Zrinka Rob, Marko Lucijanić, Laura Čmarec Buhin, Ivica Grgurević

**Affiliations:** 1 Department of Gastroenterology, Hepatology, and Clinical Nutrition, Dubrava University Hospital, Zagreb, Croatia; 2 Department of Hematology, Dubrava University Hospital, Zagreb, Croatia; 3 Zagreb University School of Medicine, Zagreb, Croatia; 4 Division for Liver Diseases, Department of Gastroenterology, Dubrava University Hospital, Zagreb, Croatia; 5 Faculty of Pharmacy and Biochemistry, University of Zagreb, Zagreb, Croatia

## Abstract

**Aim:**

To explore the prognostic value of modified Discriminant Function (mDF), Glasgow Alcoholic Hepatitis Score (GAHS), Model of End Stage Liver Disease (MELD), Age-Bilirubin-International Normalized Ratio-Creatinine score (ABIC), and the Lille Model for the 28- and 90-day mortality in patients with alcoholic hepatitis.

**Methods:**

This retrospective study enrolled patients treated for alcoholic hepatitis in Dubrava University Hospital between January 2014 and May 2018. The diagnosis was established based on histology findings or the combination of patient´s history of ongoing alcohol consumption before hospitalization, serum bilirubin above 50 mmol/L, and aspartate transaminase to alanine transaminase ratio greater than 1.5. We calculated mDF, MELD, GAHS, and ABIC on the first and seventh day of hospitalization (including the Lille model).

**Results:**

In total, 70 patients were enrolled. ABIC at admission most accurately predicted the 28-day mortality, with a cut-off of 9.92 (AUC 0.727; 95% CI 0.608-0.827, *P* = 0.0119), while GAHS most accurately predicted the 90-day mortality, calculated both at admission (cut off >7, AUC 0.765, 95% CI 0.639-0.864, *P* < 0.0001) and after seven days of hospitalization (cut-off >8, AUC 0.835 95% CI 0.716-0.918, *P* < 0.0001). Modified DF was able to predict the 28- and 90-day mortality only when calculated after seven days of hospitalization.

**Conclusion:**

There is a need for better prognostic indicators for patients with AH.

Alcoholic hepatitis (AH) is a clinical entity characterized by a sudden onset of jaundice and coagulopathy, often accompanied by elements of systemic inflammatory response syndrome, such as pyrexia and leukocytosis. About 35% of patients with alcohol-related liver disease (ALD) develop AH with steatohepatitis as the main histologic feature, whereas most patients who present with a severe form of AH (SAH) have already developed cirrhosis (1-3). High mortality rates of 16% and 30% at one and three months, respectively, with an overall five-year survival of 56%, indicate the importance of early recognition and adequate management of patients with SAH (4,5).

Historically, SAH was defined as modified Discriminant Function (mDF)≥32, a cut-off above which patients had significantly higher mortality rates and benefited from methylprednisolone therapy (6). Indeed, a recent randomized control trial on more than 1100 patients has confirmed that steroid therapy decreased the 28-day mortality in these patients. This benefit, however, was observed only in a subgroup of patients with SAH without overt sepsis or gastrointestinal hemorrhage at presentation. Furthermore, corticosteroid therapy beyond 28 days yielded no survival benefit (4). Consequently, some authors emphasized that mDF suffered from a major limitation: it is highly sensitive but not as specific. This results in the overexposure of some patients to steroid therapy and subsequent higher infection rates, without a clear therapeutic benefit (7,8).

Alternatives to mDF have shown better prognostic values (7). The Model for End-Stage Liver Disease (MELD) was originally developed (published in 2000) to predict the outcome of cirrhotic patients undergoing elective transjugular intrahepatic portosystemic shunt but was subsequently shown as an independent survival predictor in various cohorts of cirrhotic patients (9,10). It is comparable to mDF in predicting the outcome in patients with AH. Furthermore, MELD is easier to apply than mDF since it uses international standardized ratio (INR) instead of prothrombin time (PT) in seconds (11). Still, MELD has the drawback of using creatinine, which needs to be adjusted in the context of severe hyperbilirubinemia. In 2005, Forrest et al proposed the use of Glasgow Alcoholic Hepatitis Score (GAHS), a tool that was even simpler to calculate using independent factors associated with increased mortality (age, white cell count, urea, INR, and bilirubin) and that showed better results than mDF (12). GAHS has been advocated as an alternative to mDF by the updated guidelines of the European Association for the Study of the Liver, whereas the American College of Gastroenterology has advocated the use of MELD (13,14). The Lille Model (which uses age, albumin, bilirubin – initial and after 7 days, creatinine, and PT) is another prognostic tool, introduced in 2007, used to assess the efficacy of corticosteroid therapy in patients with SAH, ie, to predict poor survival in corticosteroid-treated patients (15). It was shown to outperform mDF, GAHS, and MELD in accuracy. Lastly, Age-Bilirubin-International Normalized Ratio-Creatinine score (ABIC), validated in 2008, can further stratify patients into three risk-categories with the cut-off values ranging from 6.71 to 9 (low, intermediate, and high risk of death). Dominguez et al proposed that patients with intermediate and high risk of death receive corticosteroid therapy (16). In these patients, ABIC was shown to better predict longer term (90-day) mortality than mDF (16). The aim of our study was to evaluate the prognostic value of the mentioned scoring systems in a group of AH patients from a single tertiary center.

## MATERIALS AND METHODS

This retrospective study enrolled patients with AH admitted to the Department of Gastroenterology, Dubrava University Hospital, during a 52-month period between January 2014 and May 2018. In this period, the department did not have a dedicated liver unit. Our center serves a population of around 320 000 from Zagreb and the Northwest part of Croatia.

ALD constitutes a major cause of chronic liver disease. The diagnosis of AH was established based on histological findings in the patients who had undergone liver biopsy or based on a combination of alcohol abuse, serum bilirubin above 50 mmol/L, aspartate transaminase greater than twice the upper limit of normal, and aspartate transaminase to alanine transaminase ratio >1.5 (3,13). Severe AH was defined as mDF>32. Alcohol consumption was recorded in patients´ history as reported by the patients themselves. No screening tools were used to define the exact amount. Harmful drinking was defined as more than two drinks per day for women and three drinks per day for men (17). Other causes of chronic liver disease, such as viral hepatitis, metabolic or autoimmune liver diseases, biliary obstruction, hepatic or portal vein thrombosis, and hepatocellular carcinoma, were excluded based on serological and imaging testing. From data in the hospital digital records, we calculated mDF, MELD, GAHS, and ABIC on the first and seventh day of admission for all patients regardless of corticosteroid treatment. In addition, the Lille Model was calculated on the seventh day for all patients regardless of the treatment plan. We present both standard cut-off values and custom calculated cut-off values to maximize the prognostic properties of different scores in our data set. In addition, the results of the microbiological analysis of blood, urine, stool, and ascites obtained on admission and during hospitalization were also recorded. Patients with overt sepsis or gastrointestinal bleeding and those with insufficient records were excluded from the analysis. The study was approved by Dubrava University Hospital Ethics Committee.

### Statistical analysis

The normality of distribution of numerical values was tested with the Shapiro-Wilk test, and the variables are expressed as mean ± standard deviation or as median and interquartile range (IQR). Differences in numerical values between the sexes were compared with the *t* test or Mann-Whitney U test, depending on the normality of distribution. Categorical variables are expressed as ratio and percentage, and were compared between the sexes with the Χ^2^ test. Cut-off values and diagnostic accuracy were tested with receiver operating characteristic (ROC) curve analysis. *P* values <0.05 were considered significant. The analysis was performed with MedCalc, version 18.9 (MedCalc Software bvba, Ostend, Belgium).

## RESULTS

### Patients' characteristics

Out of 284 patients treated for liver disease in our department during the four-year period, 83 were diagnosed with AH. Two patients with AH experienced variceal bleeding during hospitalization. Three patients had signs of severe infection/sepsis at admission, one patient was discharged on his own demand within five days of hospitalization, and seven patients were not enrolled due to lack of records beyond admission. Of the 70 enrolled patients, 52 (74.3%) were men. The mean age was 55.8 ± 10.7 years. In six patients, the diagnosis of AH was based on histological findings consistent with AH. The rest of the patients had clinical parameters suggestive of AH. More than a half of patients (54.2%) had comorbid illnesses, the most prevalent being cardiovascular diseases (24/38, 63.1%). The four patients in whom microbiological samples were not obtained were excluded from the infection-related analysis. Infections were documented in 9/66 (13.6%) patients on admission, and in 12/64 (18.8%) patients during hospitalization (12.9% and 17.1%, respectively, when considering the whole sample). The most prevalent type of infections were urinary tract infections, occurring in 16/21 (76.1%) of infected patients. Blood cultures were positive in 6/21 (28.5%) of infected patients. Female sex was significantly associated with developing infection during hospitalization (38.9% vs 10.9%, *P* = 0.028). The comparison of clinical parameters and demographic data between male and female patients is shown in Table 1. Score results are summarized in Table 2.

**Table 1:** The characteristics of patients stratified according to sex. The values are presented as median (interquartile range) unless indicated otherwise

	Total	Men	Women	*P^†^*
**No. of patients**	70	52	18	-
**Age; mean ± standard deviation**	55.8 ± 10.7	55.9 ± 11.3	55.3 ± 9	0.828
**Leukocytes ( × 10^9^/L)**	8.2 (5.8-12.5)	8 (6.2-12.7)	8.5 (5.6-10)	0.672
**Neutrophils ( × 10^9^/L)**	5.6 (4-9.8)	5.6 (4.4-9.9)	5.5 (3.6-7.9)	0.548
**Lymphocytes ( × 10^9^/L)**	1.2 (0.9-1.7)	1.2 (0.9-1.5)	1.4 (0.9-2)	0.504
**Platelets** **( × 10^9^/L)**	98.5 (69.3-153)	104.5 (76-153.3)	83.5 (66-132.5)	0.251
**Urea** **(mmol/L)**	4.6 (2.6-6.4)	4.6 (2.6-5.8)	4.8 (2.7-7.2)	0.830
**Creatinine** **(mmol/L)**	88 (68.8-117.5)	88.5 (67.5-118.5)	85 (71.3-113.8)	0.984
**Albumin** **(g/L)**	26.9 ± 5.6	27.5 ± 5.9	25.3 ± 4.4	0.154
**Aspartate aminotransferase** **(U/L)**	158.5 (110-272.8)	163 (121-275)	120.5 (77.5-250)	0.057
**Alanine aminotransferase** **(U/L)**	60 (39.3-94.5)	63 (44.8-107.5)	48.5 (32.3-66)	0.040
**Aspartate aminotransferase** **/alanine aminotransferase**	2.7 ± 0.9	2.7 ± 0.9	2.6 ± 0.9	0.670
**Gamma-glutamyl transferase** **(U/L)**	407.5 (86.8-1108.8)	482 (130.3-1144)	207 (53-735)	0.108
**Alkaline phosphatase** **(U/L)**	162.5 (120.8-251.8)	167.5 (126.8-260.5)	144 (110.8-222.8)	0.420
**Total bilirubin (mmol/L)**	176.5 (117.5-272)	178 (122.8-250.3)	156 (98-274.3)	0.752
**Prothrombin time (%)**	44.5 (39-55.8)	46.5 (40-57.5)	42.5 (36.3-44)	0.045
**Esophageal varices; n/N (%)**	42/67 (62.7)	31/50 (62)	11/17 (64.7)	0.842
**no varices**	25/67 (37.3)	19/50 (38)	6/17 (35.2)	
**missing data**	3/70 (4.3)	2/52 (3.8)	1/18 (5.6)	
Grade of varices; n/N (%)* **°**				0.361
**1**	17/42 (40.5)	13/31 (41.9)	4/11 (36.4)	
**2°**	21/42 (50)	14/31 (45.2)	7/11 (63.6)	
**3°**	4/42 (9.5)	4/31 (12.9)	0/11 (0)	
**Portal gastropathy; n/N (%)**	43/67 (64.2)	32/50 (64)	11/17 (64.7)	0.958
**no gastropathy**	24/67 (35.8)	18/50 (36)	6/17 (35.2)	
**missing data**	3/70 (4.3)	2/52 (3.8)	1/18 (5.6)	
**No comorbidities; n/N (%)**	32/70 (45.7)	26/52 (50)	6/18 (33.3)	0.221
**Steroid therapy; n/N (%)**	26/70 (37.1)	18/52 (34.6)	8/18 (44.4)	0.457
**Infection; n/N (%)**	21/70 (30)	14/52 (26.9)	7/18 (38.9)	0.450
**missing data**	4/70 (5.7)	4/52 (7.7)	-	
**available data**	21/66 (31.8)	14/48 (29.2)	7/18 (38.9)	
**Infection at admission; n/N (%)**	9/70 (12.9)	8/52 (15.4)	1/18 (5.6)	0.425
**missing data**	4/70 (5.7)	4/52 (7.7)	-	
**available data**	9/66 (13.6)	8/48 (16.7)	1/18 (5.6)	
**Infection during hospitalization; n/N (%)**	12/70 (17.1)	5/52 (9.6)	7/18 (38.9)	0.028
**missing data**	6/70 (8.6)	6/52 (11.5)	-	
**available data**	12/64 (18.8)	5/46 (10.9)	7/18 (38.9)	
**Death at 28 days; n/N (%)**	11/70 (15.7)	8/52 (15.4)	3/18 (16.7)	1.000
**Death at 90 days; n/N (%)**	16/70 (22.9)	10/52 (19.2)	6/18 (33.3)	0.322
**lost**	9/70 (12.9)	7/52 (13.5)	2/18 (11.1)	
**available data**	16/61 (26.2)	10/45 (22.2)	6/16 (37.5)	

*Westaby classification.

†*P* values were calculated by comparing patients with available data; *P* values <0.05 were considered significant.

**Table 2:** Score median results stratified according to sex and time of measurement. The values are presented as median (interquartile range) unless indicated otherwise*

	Total	Men	Women	P^†^
**No. of patients**	70	52	18	-
**mDF day 1**	45 (32-62.3)	41.5 (31.8-57.3)	59.5 (39.8-75.5)	0.066
**mDF day 7**	44.2 (24.5-61.1)	43 (21.2-57.9)	52.4 (35-67.2)	0.115
**GAHS day 1**	8 (7-9)	8 (7-9)	8 (8-9)	0.330
**GAHS day 7**	8 (7-9)	8 (7-9)	8 (7-9)	0.545
**MELD day 1**	21.6 (19.3-26.6)	21.5 (19.2-26)	24.5 (19.7-29.9)	0.307
**MELD day 7**	20.5 (16.5-23.9)	20.6 (16.2-23.7)	19.9 (16.6-24.9)	0.910
**ABIC day 1**	8.2 (7.2-9.1)	8.1 (7.3-9.1)	8.7 (7.2-8.9)	0.568
**ABIC day 7**	8 (7.1-9)	8 (7-9.3)	8.1 (7.5-8.7)	0.666
**Lille Model day 7**	0.4 (0.2-0.8)	0.4 (0.1-0.8)	0.4 (0.3-0.7)	0.564

*Abbreviations: mDF – modified Discriminant Function; GAHS – Glasgow Alcoholic Hepatitis Score; MELD – Model of End Stage Disease; ABIC – Age-Bilirubin- International Normalized Ratio-Creatinine score.

†*P* values were calculated by comparing patients with available data; *P* values <0.05 were considered significant.

More than two thirds of patients (53/70, 75.7%) had severe AH, with mDF>32. Of those, 26 (49%) received prednisolone treatment. Among the 27 patients with SAH not treated with corticosteroids, 44.3% had signs of infection. With regards to survival, treated patients did not significantly differ from non-treated patients (survival rates at 28 days 76.9% vs 82.7%, respectively, *P* = 0.194). Likewise, corticosteroid therapy was not associated with a significant increase in infection rates (*P* = 0.838).

### Predicting the 28-day mortality

Within 28 days of admission, 11/70 (15.7%) patients died. All deaths occurred during the hospitalization. ABIC calculated on the first day of hospitalization showed the best discriminatory properties for predicting the 28-day mortality, with a sensitivity of 54.5% and a specificity of 91.5%. The optimal cut-off value was set at 9.92 (AUC 0.727; 95% CI 0.608-0.827, *P* = 0.0119). MELD score had a similar accuracy, with slightly lower AUC 0.720 (95% CI 0.600-0.821, *P* = 0.0029) at a cut-off >21.5. GAHS was less accurate, at a cut-off >7, with AUC 0.664 (95% CI 0.541-0.773, *P* = 0.0302). Modified DF was not able to predict the 28-day mortality unless calculated on day seven.

When the tests were recalculated after seven days of hospitalization, the Lille Model most accurately predicted the 28-day mortality, with a cut-off >0.78 (AUC 0.897; 95% CI 0.798-0.958, *P* < 0.0001), sensitivity of 88.9%, and specificity of 91.4%. The second most accurate test was ABIC, with a cut-off >8.26 (AUC 0.831, 95% CI 0.721-0.911, *P* = <0.0001), followed by MELD, GAHS, and mDF. The optimal cut-off values and ROC analysis for the 28-day mortality are shown in Table 3.

**Table 3:** Receiver operating characteristic analysis of different scores for predicting the 28-day mortality. Optimal cut-off values for this sample, as well as standard ones were tested*

Prognostic score	Cut-off	No. of patients above the cut-off	No. of patients below the cut-off	Sensitivity	Specificity	Area under the curve	95% confidence interval	P value†
**mDF day 1**	>27.6	56	14	100.00	23.73	0.569	0.445-0.687	0.3817
	≥32	53	17	90.91	27.12	0.590	0.466-0.706	0.2948
**mDF day 7**	>42.7	36	34	100.00	53.45	0.758	0.639-0.853	0.0001
	≥32	46	24	100.00	37.93	0.690	0.566-0.796	0.0088
**GAHS day 1**	>7	62	8	90.91	37.29	0.664	0.541-0.773	0.0302
	≥9	24	46	54.55	69.49	0.620	0.496-0.734	0.2060
**GAHS day 7**	>8	24	46	80.00	72.41	0.812	0.699-0.897	<0.0001
	≥9	24	46	80.00	72.41	0.812	0.699-0.897	<0.0001
**MELD day 1**	>21.5	37	33	81.82	55.93	0.720	0.600-0.821	0.0029
**MELD day 7**	>22	32	38	90.00	70.69	0.856	0.750-0.929	<0.0001
**ABIC day 1**	>9.92	13	57	54.55	91.53	0.727	0.608-0.827	0.0119
	≥6.71	61	9	90.91	13.56	0.522	0.399-0.643	0.8104
	>9	19	51	54.55	77.97	0.663	0.540-0.771	0.0882
**ABIC day 7**	>8.26	30	40	90.00	67.24	0.831	0.721-0.911	<0.0001
	≥6.71	58	12	100.00	20.69	0.603	0.477-0.720	0.2236
	>9	20	50	60.00	79.31	0.697	0.573-0.802	0.0430
**Lille Model day 7**	>0.799	17	53	88.89	91.38	0.897	0.798-0.958	<0.0001
	≥0.45	40	30	88.89	62.07	0.755	0.634-0.852	0.0009

*mDF – modified Discriminant Function; GAHS – Glasgow Alcoholic Hepatitis Score; MELD –Model of End Stage Disease; ABIC – Age-Bilirubin- International Normalized Ratio-Creatinine score.

†*P* values were calculated by comparing patients with available data; *P* values <0.05 were considered significant.

### Predicting the 90-day mortality

Nine patients were lost to follow-up beyond 28 days. In this period, 16 patients died. Overall, GAHS most accurately predicted the 90-day mortality, both when calculated at admission (cut-off >7, AUC 0.765; 95% CI 0.639-0.864, *P* = 0.0001) and after seven days (cut-off >8, AUC 0.835; 95% CI 0.716-0.918, *P* < 0.0001). ABIC was more accurate than MELD (AUC 0.74 vs 0.70) on admission, and *vice versa* after seven days (AUC 0.792 vs 0.769). The Lille Model was more accurate than ABIC and MELD. Again, mDF was predictive only when calculated on the seventh day and was the least accurate score overall. The optimal cut-off values and ROC analysis for the 90-day mortality are shown in Table 4.

**Table 4:** Receiver operating characteristic analysis of different scores for predicting the 90-day mortality. Optimal cut-off values for this cohort, as well as standard ones were tested

Prognostic score	Cut-off	No. of patients above the cut-off	No. of patients below the cut-off	Sensitivity	Specificity	Area under the curve	95% confidence interval	P value^†^
**mDF day 1**	>36.2	38	23	87.50	46.67	0.635	0.502-0.755	0.0733
	≥32	45	16	93.75	33.33	0.635	0.502-0.755	0.0691
**mDF day 7**	>48.3	24	37	66.67	71.11	0.719	0.588-0.827	0.0046
	≥32	39	22	80.00	42.22	0.611	0.477-0.734	0.1694
**GAHS^†^day 1**	>7	39	22	100.00	48.89	0.765	0.639-0.864	<0.0001
	≥9	18	43	50.00	77.78	0.639	0.506-0.758	0.1002
**GAHS day 7**	>8	18	43	66.67	84.44	0.835	0.716-0.918	<0.0001
	≥9	18	43	66.67	84.44	0.835	0.716-0.918	<0.0001
**MELD day 1**	>20.7	34	27	87.50	55.56	0.700	0.569-0.811	0.0072
**MELD day 7**	>19.9	29	32	86.67	66.67	0.792	0.668-0.886	<0.0001
**ABIC day 1**	>8.12	29	32	87.50	66.67	0.743	0.615-0.846	0.0005
	≥6.71	52	9	93.75	17.78	0.558	0.425-0.685	0.4744
	>9	13	48	37.50	84.44	0.610	0.476-0.732	0.2058
**ABIC day 7**	>8.07	25	36	80.00	71.11	0.769	0.642-0.868	<0.0001
	≥6.71	48	13	100.00	26.67	0.633	0.499 -0.754	0.0734
	>9	12	49	40.00	86.67	0.633	0.499-0.754	0.1340
**Lille Model day 7**	>0.215	38	23	100.00	51.11	0.797	0.673-0.890	<0.0001
	≥0.45	24	37	73.33	73.33	0.733	0.603-0.839	0.0024

*mDF – modified Discriminant Function; GAHS – Glasgow Alcoholic Hepatitis Score; MELD –Model of End Stage Disease; ABIC – Age-Bilirubin- International Normalized Ratio-Creatinine score.

†*P* values were calculated by comparing patients with available data; *P* values <0.05 were considered significant.

## DISCUSION

The greatest clinical challenge in managing patients with severe AH is how to optimize treatment in the sense that only patients who can benefit from corticosteroid therapy are exposed to it. Our study indicates that newer scoring systems such as GAHS, ABIC, and MELD could predict the patient's outcome equally if not better than the commonly used mDF, and that all scoring systems perform better when calculated seven days after admission. Two points should be emphasized while interpreting these findings. First, we did not directly statistically compare different scores, but only indirectly assessed which score best discriminated between alive and deceased patients at specific time points through the assessment of AUC values. Second, the scores calculated on the seventh day of admission actually predicted the 21-day mortality rather than the 28-day mortality. In addition, these scores assess a developed clinical picture, which additionally aids to their accuracy.

Since its introduction in 1978, mDF has been used to stratify patients according to disease severity. In our analysis, mDF had the lowest sensitivity among the tests used. Its specificity increased, reaching statistical significance, only when it was calculated seven days after admission. These results are in line with the literature findings (6,18). Low specificity of mDF means that some patients are unnecessary exposed to steroids, which potentially increases the infection rates. With the advancement of modern supportive measures, the specificity of mDF is likely to decrease even further.

For these reasons, other scoring systems have been tested (12,15,16,19). In our study, ABIC best predicted the 28-day mortality when calculated on admission. Other models performed similarly, with small differences in diagnostic accuracy. Of note, the Lille Model outperformed ABIC since it is calculated after seven days of hospitalization. These findings are in line with other literature reports (7,20). However, our sample was too small to statistically compare the tests.

The best predictive value for the 90-day mortality was achieved by GAHS. A recent retrospective analysis of prognostic scores (MELD, ABIC, GAHS, and mDF) using data from the STOPAH trail demonstrated similar findings (7). Furthermore, the authors concluded that significantly fewer patients will be exposed to corticosteroids if baseline and seventh-day GAHS are combined, without reducing the overall survival (7).

As regard to the cut-off values, our optimized values performed slightly better than the standard cut-offs proposed in the literature. This was expected since our values are specific for this group of patients. In general, mDF, GAHS, ABIC, and Lille Model cut-off values are well established and are in line with our findings. There is still some debate as to the optimal cut-off value for MELD (hence, we tested only our optimized values), with reported values ranging from 18 to 30.5 (7,14,21). If therapy is escalated according to the recommended threshold of >21, some patients who could benefit from therapy do not get treatment (21). Our results point to the values of >21.5 on admission and >22 on day seven as optimal for predicting the 28-day mortality. The optimal values for predicting the 90-day mortality were >20.7 on admission and >19.9 on day seven.

Our group of AH patients showed the death rates of 16% at 28 days and 26% at 90 days, which are similar to those obtained in other literature reports (4). Infection rates at admission (13.6%) and during hospitalization (18.8%) were also in line with the rates reported by other authors (4,22). Yet, in our study female patients were significantly more at risk for developing infections during hospitalization. Although the published literature does not point to sex as a risk factor for infection in these patients (22), our observation might be explained by more SAH patients in the female than in the male subgroup (77.7% vs 75%) and their somewhat higher age (55.7 vs 54.9 years). However, this explanation is rather unlikely and we are unable to explain the observed difference in any other way.

Our study suffers from several limitations. First, the small number of patients resulted in low statistical power, which might have prevented some associations to reach statistical significance. Second, corticosteroid treatment was administered based on baseline mDF score, therefore non-randomly. Steroids can alter the disease course and affect the performance of the analyzed scores and results interpretation. This bias might have attenuated the prognostic properties of mDF and can explain its poor performance compared with other scores. The same phenomenon, however, affects other investigated scores proportionally to their degree of similarity to mDF. Third, a significant portion of patients with SAH (50.9%) did not receive corticosteroid therapy for various reasons. Almost half of these patients (44.3%) did not receive it because they had infections present form the start of hospitalization, but the other half were denied corticosteroids for reasons unknown to us. Forth, we were not able to retrieve the data regarding alcohol consumption before hospitalization. Lastly, the retrospective nature of the study based on a single institution registry and experience from one tertiary center makes our findings not generalizable to ambulatory or AH patients treated in other types of hospitals.

In line with our results, we can conclude that in managing patients with AH, newer prognostic tools, such as GAHS, MELD or ABIC could have the same, if not greater, diagnostic accuracy than mDF. Nevertheless, all the scoring systems showed only modest prognostic value (depicted by low AUC), especially at admission, which puts their adequacy in determining clinical outcome and necessity for steroid therapy into question. Obviously, there is a need for a clinical tool that could better predict mortality and serve as a better guide when deciding on steroid therapy administration in patients with AH.
